# Is There a Difference in Microbiological Epidemiology and Effective Empiric Antimicrobial Therapy Comparing Fracture-Related Infection and Periprosthetic Joint Infection? A Retrospective Comparative Study

**DOI:** 10.3390/antibiotics10080921

**Published:** 2021-07-29

**Authors:** Markus Rupp, Susanne Baertl, Nike Walter, Florian Hitzenbichler, Martin Ehrenschwender, Volker Alt

**Affiliations:** 1 Department for Trauma Surgery, University Medical Center Regensburg, 93053 Regensburg, Germany; susanne.baertl@ukr.de (S.B.); nike.walter@ukr.de (N.W.); volker.alt@ukr.de (V.A.); 2 Department of Infection Prevention and Infectious Diseases, University Medical Center Regensburg, 93053 Regensburg, Germany; florian.hitzenbichler@ukr.de; 3 Institute of Clinical Microbiology and Hygiene, University Medical Center Regensburg, 93053 Regensburg, Germany; martin.ehrenschwender@ukr.de

**Keywords:** empiric antimicrobial therapy, fracture-related infection, prosthetic joint infection, difficult to treat pathogens

## Abstract

This study aims to investigate (1) microbial patterns in fracture-related infections (FRIs) in comparison to microbiological patterns of periprosthetic joint infections (PJIs), (2) the identification of effective empiric antibiotic therapy for FRIs and PJIs and (3) analysis of difficult-to-treat (DTT) pathogens. Patients treated for FRIs or PJIs from 2017 to 2020 were evaluated for pathogens detected during treatment. Antibiotic susceptibility profiles were examined with respect to broadly used antibiotics and antibiotic combinations. Resistance rates to rifampicin or fluoroquinolone were determined. A total of 81 patients with PJI and 86 with FRI were included in the study. For FRIs *Staphylococcus aureus* was the most common infection-causing pathogen (37.4% vs. 27.9% for PJI). Overall, there was no statistical difference in pathogen distribution (*p* = 0.254). For FRIs, combinations of gentamicin + vancomycin (93.2%), co-amoxiclav + glycopeptide and meropenem + vancomycin (91.9% each) would have been effective for empiric therapy, similar to PJIs. Difficult to treat pathogens were more frequently detectable in PJIs (11.6% vs. 2.3%). Empiric therapy combinations such as gentamicin + vancomycin, co-amoxiclav + glycopeptide or meropenem + vancomycin, are effective antibiotic strategies for both FRI and PJI patients. More DTT pathogens were detectable in PJIs compared to FRIs.

## 1. Introduction

Implant-associated infections, such as periprosthetic joint infection (PJI) and fracture-related infection (FRI), represent a major complication in orthopedic and trauma surgery with a significant socioeconomic burden [1,2]. Steadily increasing primary arthroplasty procedures will further boost the importance of implant-associated infections [1,3]. Understanding biofilm formation on non-living surfaces as a key element in implant-associated bone infection helped to establish curative treatment strategies, which entail both surgical management and antibiotic therapy [4]. Surgical strategies range from implant retention in case of stable implants and acute infection (which is deemed to occur within 3 weeks of symptoms) to implant exchange in a one-stage, two-stage or even multi-stage surgical treatment concept when infection is chronic or implants are loosened. Surgical approaches are usually complemented with antibiotic therapy for at least 6 weeks [5]. Initial empiric antimicrobial treatment with a glycopeptide antibiotic and a carbapenem is currently recommended for PJI to cover commonly encountered pathogens, such as methicillin-resistant (and thus, beta lactam-resistant) coagulase-negative staphylococci (CoNS), *Staphylococcus aureus* and gram-negative bacteria [6,7]. For FRI, recommendations regarding empiric antibiotic treatment have also been developed [8]. Whether the microbiological epidemiology of FRI is similar to or differs from PJI is unknown, but this knowledge is important for adequate empirical antibiotic treatment. Therefore, the purpose of this study was to answer the following questions: (1) What is the microbial epidemiology in a retrospective cohort treated for FRI? (2) Do the evidenced pathogens in FRI patients differ compared to a cohort treated at the same center for PJI? (3) What is the best possible antibiotic treatment for those FRI and PJI cases? (4) How many cases involved difficult-to-treat (DTT) pathogens in FRI and PJI patients?

## 2. Results

### 2.1. Demographics

In total, 81 patients were diagnosed with PJI. Overall, 48 (59.3%) of the patients were male and 35 (40.7%) female. Mean age was 70.6 ± 10.3 years. The mean BMI was 31.2 ± 8.2 kg/m^2^. Most patients had comorbidities with a mean CCI of 2 (range 0–6) and a mean ASA score of 3 (range 0–4). PJI mainly occurred at the knee (*n* = 42, 51.9%) and the hip (*n* = 34, 42.0%). In 19 cases (23.5%), PJI involved a revision prosthesis. The mean delay from prosthesis implantation to revision surgery due to PJI was 3.95 years (range: 9 days–29 years). The FRI cohort comprised 86 patients. Out of these, 62 (71.1%) were male and 24 (28.9%) female. The mean age was 55.9 ± 16.7 years and the mean BMI 27.5 ± 5.5 kg/m^2^. The mean CCI was 1 (range 0–6) and the mean ASA score was 2 (range 1–4). FRI mainly occurred at the tibia (43.0%), followed by infections of ankle and foot fractures (16.3% each). Initially, 19 patients (22.1%) received fracture treatment due to an open injury. The mean delay from initial fracture care to onset of infection symptoms was 0.39 years (range 3 days–9.6 years) (Table 1). In comparison, the groups did not differ significantly in gender (*p* = 0.085). However, PJI patients were older than FRI patients (*p* < 0.001), showed a higher BMI (*p* = 0.002) and had statistically significant more comorbidities as indexed by the CCI (*p* < 0.001), although not according to the ASA score (*p* = 0.085).

### 2.2. Microbiological Patterns

**Figure 1:**
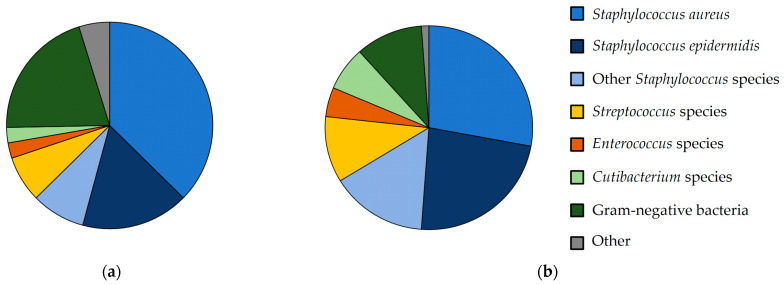
Isolated microorganisms shown in percentage from: (**a**) FRI and (**b**) PJI patients.

In 11 cases (13.6%) of the PJI patients and in 12 cases (14.0%) of the FRI patients, the infection was culture-negative. A polymicrobial infection was present in 14 cases (17.3%) of the PJI cohort and in 9 cases (10.5%) of the FRI cohort (Table 1). Isolated microorganisms of both cohorts are presented in Table 2 and Figure 1. Methicillin-sensitive *Staphylococcus aureus* was the most frequently detected pathogen in both cohorts (27.9% PJI vs 37.4% FRI). One *Staphylococcus aureus* was methicillin-resistant in the FRI cohort (1.2%). The second most evident pathogen in the PJI cohort was *Staphylococcus epidermidis* (23.3%), followed by other *Staphylococcus* species (15.1%), *Streptococcus* species and gram-negative bacteria (10.5% each). In the FRI cohort, the second most prevalent bacteria were gram-negative bacteria (20.5%), followed by *Staphylococcus epidermidis* (16.9%). A chi-square test was used to compare pathogen distribution between the two cohorts, resulting in no statistically significant difference (χ²(6) = 7.784, *p* = 0.254, φ = 0.214).

### 2.3. Antimicrobial Regimes

**Figure 2:**
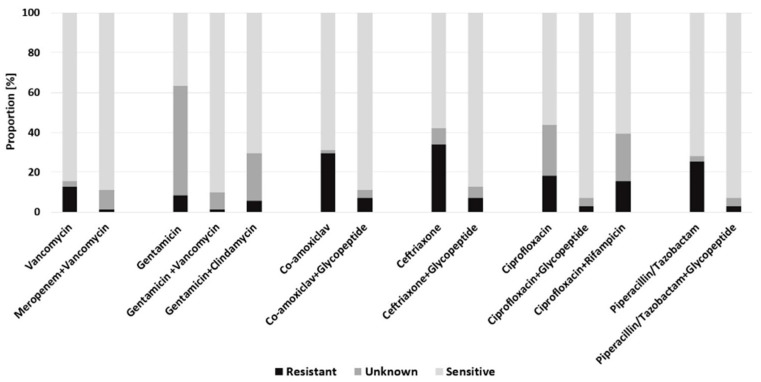
Predicted efficacy of empiric antimicrobial regimens for the PJI cohort.

To assess the best possible empirical antibiotic coverage, combinations of antibiotics have been hypothesized as a potential empirical antibiotic therapy against later evidenced pathogens. In the PJI cohort, a combination of piperacillin/tazobactam and ciprofloxacin achieved a 93.0% antibiotic coverage of later evidenced pathogens. The combination gentamicin and vancomycin would have covered 90.1% of all patients who suffered from PJI. High resistance rates were found for empirical monotherapy with ceftriaxone (33.8%), co-amoxiclav (29.6%) and piperacillin/tazobactam alone (25.4%) (Figure 2).

**Figure 3:**
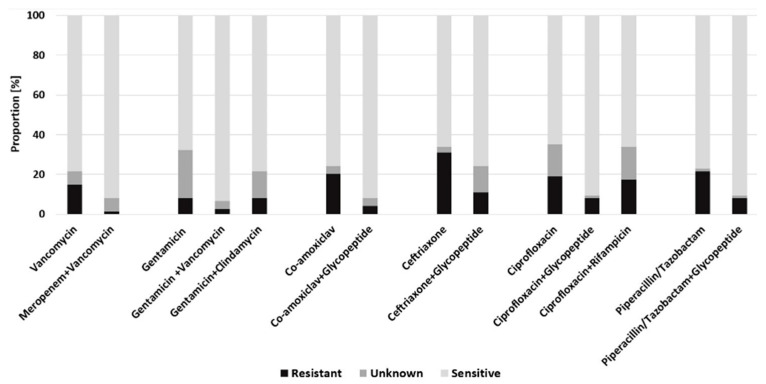
Predicted efficacy of empiric antimicrobial regimens for the FRI cohort.

In the FRI cohort, the hypothetical combination of gentamicin with vancomycin resulted in the broadest antibiotic coverage (93.2% of all patients), followed by co-amoxiclav in combination with glycopeptide antibiotics and meropenem combined with vancomycin (91.9% each). Furthermore, ciprofloxacin and piperacillin/tazobactam in combination with a glycopeptide reached an antibiotic coverage of 90.5%. For ceftriaxone (31.1%) monotherapy antibiotic coverage would have been achieved in only 31.1%, which could be improved to 10.8% by an additional combination with a glycopeptide (Figure 3). Comparing the predicted efficacy of empiric antimicrobial regimens between PJI and FRI patients only revealed a statistically significant difference regarding the combination gentamicin with vancomycin (U = 1877.00, Z = −3.326, *p* = 0.001).

## 3. Discussion

The present cohort study compares for the first-time microbiologic epidemiology of PJI with FRI at a center specialized in bone and joint infection treatment. Based on antibiotic susceptibility testing of the evidenced germs, common treatment regimens for empirical antibiotic treatment have been investigated and best practice options have been evaluated based upon the present results. For both PJI and FRI, antibiotic combinations of either meropenem + vancomycin, co-amoxiclav + glycopeptide or piperacillin/tazobactam + glycopeptide and vancomycin + gentamicin seem reasonable combinations to achieve an approximate 90% safe susceptibility to empirical antibiotic therapy.

### 3.1. Polymicrobial Infections, Culture-Negative Infections and Evidenced Pathogens in PJI and FRI

For both PJI and FRI the number of culture-negative infections and polymicrobial infections was relatively low but comparable to each other. In the literature, higher rates of polymicrobial have been reported for PJI (16–46.6%) [9,10]. In infected nonunions, which represent FRI with cessation of bone healing for more than six months after fracture, a similar rate of polymicrobial infection has been reported (14.3%) [11]. Moreover, higher rates of polymicrobial infections in FRI have been described comparing different sampling protocols (25% and 36%, respectively) [12]. The present rate of culture-negative PJIs (13.6%) was similar to previous reports indicating a proportion of 5% to 12% [13,14]. Differences in reported polymicrobial and culture-negative PJI rates might be due to local epidemiology and different diagnostic criteria used for PJI and FRI as well as previous antibiotic treatment, which decreases microbial diagnostic yield [15,16]. Reported rates for culture-negative FRIs range from 6.1% to 18.1% [17,18]. *Staphylococcus aureus* followed by *Staphylococcus epidermidis* were the most common evidenced pathogens causing FRI and PJI in the present study. This is in line with several studies investigating infection-causing agents [11,17,19]. Although no statistically significant difference could be found comparing pathogens causing PJI with pathogens evidenced in FRI, a higher rate of *Enterococcus species* and gram-negative bacteria in FRI might be due to a higher rate of previous antibiotic treatment against mainly gram-positive germs. However, this hypothesis cannot be proven due to the retrospective study design.

### 3.2. Empirical Antibiotic Therapy Regimens

There are limited data on the usefulness and adequate administration of empiric antibiotic therapy for bone and joint infections. For FRI, the inferiority of late-onset targeted antibiotic therapy has been demonstrated compared to early empiric antibiotic therapy using vancomycin combined with rifampicin [20]. However, the study included only acute FRIs in which implant retention was performed and both different antibiotic therapy protocols were compared retrospectively. Further evidence for empiric antibiotic therapy in FRI is scarce. Recommendations for empirical antibiotic therapy are based on experiences made with the treatment of PJI or osteomyelitis [8,21,22]. The present results indicate a broad coverage for vancomycin + carbapenem, vancomycin + gentamicin, piperacillin/tazobactam + glycopeptide as well as co-amoxiclav + glycopeptide for both PJI and FRI. The findings correspond with recently published findings for chronic osteomyelitis recommending carbapenems against *Pseudomonas* spp. and a glycopeptide to cover gram-positive germs [21]. Targeted antibiotic therapy should be initiated as soon as evidenced pathogens and their susceptibility are available. Since any broad-spectrum antibiotic combination encompasses the risk to facilitate antimicrobial resistance, which poses a global health care problem, de-escalation of antibiotic therapy seems reasonable as soon as causing pathogens and their antimicrobial susceptibility are identified. In addition to the importance of antibiotic stewardship to avoid antimicrobial resistance, antibiotic de-escalation should be initiated as soon as possible to prevent antibiotic-associated secondary diseases [23]. Another feasible approach to bypass unwanted side effects of systemic antibiotics is the administration of local antibiotic carriers. Based on the outlined results, the application of gentamicin + vancomycin as a commonly used local antibiotics combination achieves high coverage of up to 90.1% in PJI and 93.2% in FRI. Meanwhile, resistance against the combination of gentamicin + vancomycin is low (FRI = 2.7%, PJI = 1.4%). Based on the present data, surgical application of vancomycin + gentamicin carrying PMMA bone cements or other bone substitutes seems reasonable [24,25]. The authors prefer local administration of the combination of vancomycin + gentamicin as antibiotic combination since side effects, such as nephrotoxicity and ototoxicity, which may result from systemic standard administration of vancomycin + gentamicin, seem better to be avoided using other effective antibiotic combinations. Nevertheless, the benefits and possible downsides of clinical application have to be further investigated and surely rely on several factors, including the duration of infection, biofilm formation and surgical treatment strategies, such as implant retention and soft and bone tissue defects as well as comorbidities of the patient [26,27].

### 3.3. Difficult-to-Treat Infections

The reported numbers of DTT pathogens in PJI in this study (11.6%) is similar to previously reported rates in PJI (18.4%) [28]. For FRI, no data reporting on DTT pathogens are available in the literature. Unfortunately, in a high rate of evidenced bacteria, no testing for rifampicin or fluoroquinolones was available for the present analysis. Since biofilm formation on orthopedic implants might impede sufficient fracture healing in FRI and long-time survival of an endoprosthesis after PJI, the authors see a need for standardized evaluation of rifampicin and fluoroquinolone-resistance in routine microbiological diagnostics for implant-associated infections. Furthermore, future high-quality studies should outline the relevance of DTT pathogens for clinical outcome in terms of reinfection. For PJI, similar results comparing DTT infections with non-DTT PJIs were reported at a cost of longer hospital stay, longer prosthesis-free intervals and longer antibiotic treatment in the DTT pathogen cohort [28]. Future studies should outline the relevance of DTT pathogens for implant associated infections, which might be at least as relevant as multi-drug-resistant bacteria, which are often amenable to biofilm-active antibiotic agents.

### 3.4. Limitations

This study has some limitations. Data analysis of one orthopedic center may lead to a local epidemiological bias. In addition, the retrospective design restricts analysis to already existing resistograms. In some cases, antibiotic testing for certain antibiotics was not performed, which leads to “unknown” listed antibiotic susceptibility. This is mainly due to different panels of antibiotics available for automated and manual susceptibility testing according to the interpretative criteria released by the European Committee on Antimicrobial Susceptibility Testing (EUCAST).

In addition, the retrospective file analysis did not consistently allow identification of antibiotic pretreatment and its effect on the detection of infection-causing pathogens. The analysis of PJI and FRI patients from 2017 to 2020 ensures consistent diagnosis and inclusion of patients according to well-established diagnostic criteria. However, subgroup analysis to answer questions concerning the relevance of anatomic location or durability of infection was not possible due to the low volume in numbers.

## 4. Materials and Methods

### 4.1. Patient Identification

A retrospective cohort study of patients treated for FRI or PJI was conducted in a level 1 trauma center in Germany. Prior of beginning the study, a positive ethics committee votum was obtained from the ethics committee of the University Hospital Regensburg (file number 20-1680-101). The inclusion period was defined from 1 January 2017 to 31 December 2020. Eligible patients being 18 years or older were screened by international classification of disease (ICD)-10 diagnosis codes (T84.5 infection and inflammatory reaction due to internal joint prosthesis, T84.6 infection and inflammatory reaction due to internal fixation device). Afterwards, patients’ medical charts, surgery protocols, laboratory findings as well as microbiological and histopathological reports were screened for inclusion criteria of PJI and FRI. PJI was considered confirmed if at least one of the following criteria was met according to the EBJIS consensus for the diagnosis of PJI [15]. FRI was defined according to the definitions of the FRI consensus group published in 2018 [29]. Patients were enrolled regardless of whether they presented with primary infection or reinfection. Furthermore, patients presenting with culture-negative infections were included. If deep tissue samples or synovial fluid were not collected for microbiological analysis, patients were excluded for analysis (*n* = 4). Difficult-to-treat FRI or PJI were present when at least one of the causing microorganisms was resistant to biofilm-active antibiotics including rifampicin-resistant staphylococci, enterococci, fluoroquinolone-resistant gram-negative bacteria and fungi [30].

### 4.2. Data Collection

Patient characteristics (sex, age and BMI at the time of surgery) and details of orthopedic implant-associated infections (site of infection or index joint, type of implant or arthroplasty and reinfection) were assessed retrospectively by reviewing electronic medical records. Comorbidities were assessed by obtaining the ASA score as well as the Charlson Comorbidity Index (CCI). The microbiological database was searched for the pathogens detected and for antimicrobial susceptibility testing. Detection was either based on preoperatively or intraoperatively deep tissue sampling or aspiration of the affected joint. Routinely, at least five deep tissue samples have been taken for microbiological analysis.

### 4.3. Microbiology

All tissue samples collected were homogenized and seeded on solid and liquid culture media. Agar plates were incubated for 48 h in the absence and presence of CO_2_. Liquid culture media were incubated for 14 days in ambient air. All culture media were incubated at 37 °C and inspected every day for bacterial growth. Bacteria were identified by matrix-assisted laser desorption ionization time of flight mass spectrometry (MALDI TOF MS) using a Microflex LT mass spectrometer and BioTyper software (Bruker Daltonics, Bremen, Germany). Antibiotic susceptibility testing followed guidelines from the European Committee on Antimicrobial Susceptibility Testing (EUCAST) and was performed using a BD Pheonix M50 nephelometer or manually by disc diffusion. In case of removal of orthopedic devices, sonication of the orthopedic implants has been performed, as mentioned before [31].

### 4.4. Statistics

Descriptive and statistical data analysis was performed using the IBM SPSS Statistics software (version 24.0, IBM Corp, Armonk, NY, USA). Frequencies were expressed as numbers and percentages. Continuous parameters were presented as means ± standard deviation (SD) and compared by Student’s *t*-test. Chi-square test was used for comparison of categorical variables. Mann–Whitney-U-test was calculated to determine if there were differences in the antimicrobial regimes between PJI and FRI patients. For all tests, *p* values < 0.05 were considered statistically significant.

## 5. Conclusions

Despite the undoubted differences between PJIs and FRIs, which range from etiopathogenesis to possible therapeutic strategies, the pathogens that cause infection in both diseases are comparable. The hypothetical analysis of possible antibiotic regimes reveals that for an empirical antibiotic therapy, a combination of vancomycin + meropenem, glycopeptide + co-amoxiclav or piperacillin/tazobactam + glycopeptide are the best therapy options for both FRIs and PJIs. For local antibiotics, the well-established combination of vancomycin with gentamicin is the best treatment option. Difficult-to-treat bacteria are more often evidenced in PJI than FRI. Standardized microbial testing against biofilm active antibiotics such as rifampicin and fluoroquinolones should be implemented in routine microbiological testing for implant associated infections.

## Figures and Tables

**Table 1:** Baseline characteristics of the cohorts consisting of PJI and FRI patients.

Characteristic	PJI (*n* = 81)	FRI (*n* = 86)
Demographic data		
Sex (male)	48 (59.3%)	62 (71.1%)
Age (years)	70.6 ± 10.3	55.9 ± 16.7
BMI (kg/m^2^)	31.2 ± 8.2	27.5 ± 5.5
ASA score	3 [–4]	2 [1–4]
CCI	2 [0–6]	1 [0–6]
Site		
Hip	34 (42.0%)	11 (12.8%)
Knee	42 (51.9%)	6 (7.0%)
Shoulder	5 (6.2%)	2 (2.3%)
Forearm		38 (44.2%)
Tibia		14 (16.3%)
Ankle		14 (16.3%)
Foot		1 (1.2%)
Revision prosthesis	19 (23.5%)	
Delay from prosthesis implantation to surgery/delay from trauma to infection	3.95 years [9 days–29 years]	0.39 years [3 days–9.6 years]
Microbiologic documentation		
Negative culture	11 (13.6%)	12 (14.0%)
Polymicrobial infection	14 (17.3%)	9 (10.5%)

**Table 2:** Isolated microorganisms.

	PJI (*n* = 86)	FRI (*n* = 83)
*Staphylococcus aureus*	24 (27.9%)	31 (37.4%)
*Staphylococcus epidermidis*	20 (23.3%)	14 (16.9%)
Other *Staphylococcus* species	13 (15.1%)	7 (8.4)
*Streptococcus* species	9 (10.5%) 6 *Streptococcus dysgalactiae* 3 *Streptococcus agalactiae*	6 (7.2%) 5 *Streptococcus dysgalactiae* 1 *Streptococcus agalactiae*
*Enterococcus* species	4 (4.7%) 4 *Enterococcus faecium*	2 (2.4%) 2 *Enterococcus faecium*
*Cutibacterium species*	6 (7.0%)	2 (2.4%)
Gram-negative bacteria	9 (10.5%) 4 *Pseudomonas aeruginosa* 2 *Escherichia coli* 1 *Enterobacter cloacae* 1 *Aeromonas bestiarum* 1 *Bacteroides fragilis*	17 (20.5%) 3 *Pseudomonas aeruginosa* 5 *Escherichia coli* 1 *Proteus hauseri* 5 *Enterobacter cloacae* 1 *Citrobacter gillenii* 1 *Serratia marcescens* 1 *Methylorubrum populi*
Other	1 (1.2%) 1 *Kocuria rhizophila*	4 (4.8%) 3 *Bacillus cereus* 1 *Clostridium subterminale*

## Data Availability

The datasets generated and analyzed in the current study are available from the corresponding author on reasonable request.
